# The reemergence of human rabies and emergence of an Indian subcontinent lineage in Tibet, China

**DOI:** 10.1371/journal.pntd.0007036

**Published:** 2019-01-14

**Authors:** Xiao-Yan Tao, Mu-Li Li, Qian Wang, Ciwang Baima, Mei Hong, Wei Li, Yong-Biao Wu, Yan-Rong Li, Yu-Min Zhao, Simon Rayner, Wu-Yang Zhu

**Affiliations:** 1 Key Laboratory for Medical Virology, Ministry of Health, National Institute for Viral Disease Control and Prevention, Chinese Center for Disease Control and Prevention, Beijing, China; 2 Department of Pathology, Shenzhen People’s Hospital, Shenzhen, Guangdong, China; 3 Tibet Center for Disease Control and Prevention, Lhasa, Tibet, China; 4 Sichuan Center for Disease Control and Prevention, Chengdu, Sichuan, China; 5 Wuhou District Center for Disease Control and Prevention, Chengdu, Sichuan, China; 6 Department of Parasitology, Guilin Medical University, Guilin, Guangxi; 7 Department of Medical Genetics, Oslo University Hospital and University of Oslo, Oslo, Norway; 8 Hybrid Technology Hub - Centre of Excellence, Institute of Basic Medical Sciences, University of Oslo, Oslo, Norway; Wistar Institute, UNITED STATES

## Abstract

Coordinated surveillance, vaccination and public information efforts have brought the Chinese rabies epizootic under control, but significant numbers of fatalities are still reported annually with some cases occurring in previously rabies free regions. Tibet has remained virtually rabies free for 16 years, but since 2015 one human rabies case has been reported each year. To better understand the origins of these cases, we sequenced three human samples and an additional sample isolated from a dog in 2012. Three genomes were sequenced from brain samples: human case 1 (reported in 2015), human case 3 (2017), and the 2012 dog case. For human case 2 (2016), the rabies N gene was sequenced from a limited saliva sample. Phylogenetic analysis shows that Case 1 (CXZ1501H) and the dog case (CXZ1201D) belong to China IV lineage (equivalent to Arctic-like-2 in global rabies), suggesting an association with a wildlife spillover event. However, Case 2 (CXZ1601H) is placed within the dominant lineage China I, and was most similar with recent strains from neighboring Yunnan province, indicating the current epizootic has finally reached Tibet. Most surprisingly however, was the finding that Case 3 (CXZ1704H) is distinct from other Chinese isolates. This isolate is placed in the Indian Subcontinent clade, similar to recent Nepal strains, indicating that cross-border transmission is a new source for rabies infections. Thus, the complex mixture of the rabies epizootic in Tibet represents a major new challenge for Tibet and national rabies control.

## Author summary

Until recently, Tibet, “the third pole” of the earth, has been relatively isolated from the outside world, and has stayed rabies free for almost two decades. However, from 2015 to 2017, one human case has been reported each year. Investigation of the origins of these cases revealed each of the three human cases has distinct origins. Case 1 (2015) seems to be a result of a wildlife spillover event, and is consistent with rabies virus strains found in Russia, and Qinghai, Inner Mongolia and Heilongjiang in China. Case 2 (2016) is associated with the current Chinese epizootic and appears to have originated from the neighboring province of Yunnan. Lastly, Case 3 (2017) is most closely related to a few recent Nepalese strains and originates from the Indian subcontinent clade, also found in India and Sri Lanka. Thus, Tibet is facing a major rabies threat on three fronts.

## Introduction

China has been facing an ongoing rabies epizootic since the middle of the 1990s [1–3] that, consistent with many other countries experiencing major extended rabies outbreaks [4] is primarily spread by domestic dogs transmitting the rabies virus (RABV)[1,3], a species of the Genus *Lyssavirus* [4–6].

China is a large country with a diverse geography, climate and demographics and, consequently, the burden of rabies varies by region. The southern and eastern parts of the country report the majority of human cases [3,7], but almost all provinces have experienced some degree of impact from the disease. Therefore, to reduce rabies fatalities and bring the epizootic under control, the government established a comprehensive surveillance program and investigated the efficacy of different control methods such as post exposure treatment, vaccination and education[7]. As a result, the number of cases has steadily decreased in the last ten years [3,7,8]. However, the number of provinces reporting cases has slowly expanded to encompass most of the country, with sporadic events even reported in regions in western China, which had previously been rabies free for more than 20 years [9].

Investigation of the diversity and evolution of viruses over the course of an epidemic can help in the development of strategies to combat and control viral diseases [1,10,11]. Based on our continued nationwide surveillance and other Chinese reported rabies studies, the Chinese street strains of RABV can be divided into six major lineages [1,12]. In the context of global RABV clades, China I, China II, China V and China VI are sub-clades of the Asian clade; China III corresponds to Cosmopolitan clade; and China IV corresponds to Arctic-like-2 in the Arctic-related clade [1,9,13,14]. Among these lineages, China I has emerged to become the dominant lineage in the current rabies epizootic [1], and has expanded into most provinces in mainland China, including western provinces such as Ningxia, and Gansu [9,15]. At the same time, sporadic human and dog cases have been reported in Qinghai and Tibet, but our earlier analysis of these samples indicates they belong to the China IV sub-clade, suggesting they are associated with spillover from wildlife, rather than the main epizootic [9].

A human case was reported in Tibet in 2015[16], after a 16 year hiatus and since then, additional cases have been reported[8], with the most recent case reported in 2017. As the national reference laboratory for China rabies surveillance, our lab received brain samples from both cases and these were confirmed by direct immunofluorescence assay (DFA). These two human samples, and a dog specimen also reported in Tibet in 2012 with no associated human cases [9], were subjected to whole genome sequencing and phylogenetic analysis. In addition, a further suspected Tibetan human case reported in 2016 was subsequently confirmed by the Sichuan provincial CDC and the N gene of the isolate was sequenced and analyzed.

## Materials and methods

### Ethics statement

The program for collection of human brain specimens was approved by the Ethical Committee of the National Institute of Viral Disease Control and Prevention, China CDC, which is the national referral center for rabies diagnosis. Due to their medical condition, subjects were unable to provide consent once a rabies infection was suspected and so written informed consent was obtained in both cases from their relatives after death.

### Epidemiological data

Data on Tibet human rabies cases were collected from the Chinese Notifiable Disease Reporting System (NDRS) of the China CDC [3]. The reporting methods and how cases were determined to be associated with rabies were the same as described previously [3,17]. More detailed epidemiological information of the cases was collected by the local CDCs using a standard report format.

### Specimen collection and detection

From 2015 to 2017, a total of three human cases were reported in Tibet, one each year. Specimens of each case were collected before or after death (Table 1). The first reported human case, named Case 1, was collected as a brain sample after death and sent to the China CDC rabies laboratory for diagnosis.

**Table 1:** Specimen details of the three human cases from Tibet.

Code Name	Year	Date			type of specimen
		onset	Death	specimen collection	
Case 1	2015	10 Sep	16 Sep	17 Sep	Brain
Case 2	2016	10 Jul	26 Jul	18 Jul	Saliva, CSF
Case 3	2017	19 Jan	23 Jan	21 Jan	Saliva, Urine, Serum
				23 Jan	Brain, Skin at the nape of the neck

The second case, named Case 2, was transferred to a hospital in Sichuan from Tibet on July 15, 2016, 5 days after the onset of the disease. The saliva and cerebrospinal fluid (CSF) samples were collected and detected by Sichuan CDC.

Samples for the third case, named Case 3, were collected from saliva, urine and serum before death, and the brain and skin at the nape of the neck samples were collected after death. All of the samples were sent to the China CDC rabies laboratory by the Tibet CDC.

The brain tissue and the neck skin specimens were tested for rabies using DFA as described previously [18], and showed positive results, confirming the presence of rabies in Case 1 and Case 3. For the liquid specimens from Case 3, nested-PCR was used for detection as described previously [19], and all of them returned negative results. The saliva and CSF samples of Case 2 were tested using real-time PCR by the lab of Sichuan CDC [20] with a rabies virus nucleotide detection kit (DAAN GENE, Guangzhou, China), and the saliva and CSF samples tested positive and negative, respectively.

### Sequencing

The brain tissues of Case 1(named CXZ1501H) and Case 3 (named CXZ1704H) were then used to amplify the rabies genome. Total RNA extraction, cDNA synthesis and sequencing were performed using the same protocols as described previously [19,21]. In addition, genome sequencing was also performed on a dog brain specimen collected in Tibet in 2012 (CXZ1201D) as reported previously for the nucleoprotein (N) gene [9]. The full length of CXZ1501H and CXZ1201D are 11927bp, CXZ1704H is 11925bp. The saliva of Case 2 (named CXZ1601H) was used to amplify the N gene of RABV as described previously [9], since insufficient volume was available to amplify the rabies genome.

### Phylogenetic analysis

To investigate the lineages of the isolates on a national and global scale, a reference dataset was created comprising: the three new Tibetan human strains (Case 1, Case 2 and Case 3), the 2012 Tibetan dog strain and 41 representative reference N sequences of both Chinese street strains and worldwide strains (S1A Table). A genome reference dataset (S1B Table), comprising two human Tibetan strains (Case 1 and Case 3) and the 2012 dog sample plus domestic and global strains [22], was also created for comparative purposes. Multiple alignments for both datasets were produced using the Clustal X v2.1 program [23]. Phylogenetic reconstruction was performed on each dataset using the MEGA7 software package with the Neighbour-Joining (NJ) method and 1000 bootstrap replicates [24]. The NJ method was used here as the goal was simply to determine the major lineage (as opposed to making any predictions related to most recent common ancestor, or mutation rate) of the new Tibetan strains relative to (i) the six major rabies lineages, China I to China VI, that have been observed in China and (ii) the four major global rabies lineages.

### Accession numbers

Genome sequences were submitted to GenBank, with accession numbers KY175229 (case 1), KY175230 (case 3) and MH671332 (dog specimen). The accession number of the N gene from Case 2 is MH746442.

## Results

### Human rabies cases in Tibet

Tibet is located in the Qinghai-Tibet Plateau, a sparsely populated and geographically remote region with climatic extremes, and relatively isolated from the outside world. Since the 1990’s, Tibet has reported few human rabies cases (one case in 1992 and two cases in 1998), and has remained human rabies free for the last 16 years (1999–2014) [9]. However, since 2015 one human rabies case has been reported each year.

**Fig 1:**
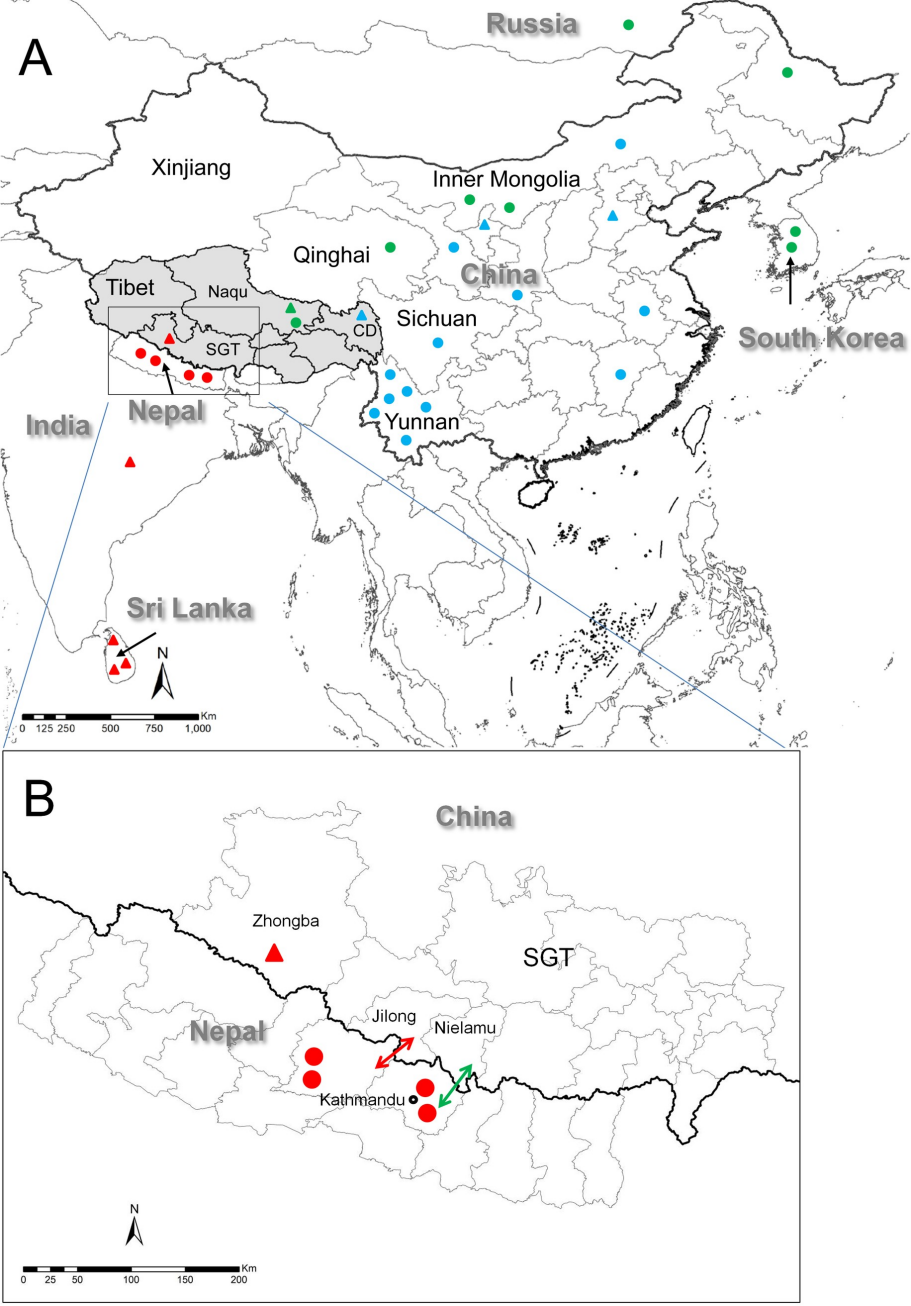
**Geographical location of recent human (2015, 2016 & 2017) and dog rabies cases (2012) in Tibet (A), and detailed map of the selected regions of China and Nepal (B).** The cities of Tibet are abbreviated as follows: SGT, Shigatse; CD, Changdu. Human cases are shown as triangles, animal cases as circles. Blue triangles or circles indicate the strains of China I lineage in Fig 2; Green triangles or circles indicate the strains of China IV / Arctic-like-2 lineage in Fig 2; Red triangles or circles indicate the strains of China VII / Indian subcontinent in Fig 2. The red two-way arrow in Fig 1B marks the Jilong customs port between China and Nepal, and the green two-way arrow marks the Zhangmu customs port. The map is derived from a Topographic Database of the National Fundamental Geographic Information System (NFGIS) from the Chinese National Administration of Surveying, Mapping and Geoinformation (http://www.tianditu.com/).

Case 1: On September 16, 2015, a young male herdsman, from Yongqu village in Nierong County, Naqu Prefecture, Tibet (Fig 1) died in the local hospital. He had exhibited clinical symptoms consistent with rabies 6 days previously. He was bitten on his left wrist by a stray dog approximately two months earlier, and he washed the wound but did not seek vaccination after the event. Consequently, this patient was reported as a suspected rabies case to the NDRS of China CDC. As this represented the possibility of the first human rabies case in Tibet in 20 years, the following day the family agreed to collection of his brain tissue, which was sent to the China CDC rabies laboratory (our lab) for laboratory diagnosis and subsequently confirmed as rabies positive.

Case 2: A young man, living in Karuo District, Changdu City in Tibet (Fig 1) began to show clinical symptoms on July 10, 2016 and went to a local hospital. On July 15, the patient was transferred to West China Hospital, in Sichuan province, and his saliva and CSF samples were collected and submitted to Sichuan CDC on July 18. The samples were investigated by Real-time PCR and the saliva sample was confirmed positive on the next day. The patient had been in contact with dogs, but had no biting injury in recent years, and died on July 26.

Case 3: On January 21, 2017, Zhongba County CDC, in Shigatse city, Tibet, received a call reporting a suspected rabies case from Payang town health center (Fig 1). The CDC staff went to Payang to obtain more details and collected the saliva, urine, and serum specimens of the patient. The patient was a herdswoman, 39 years old, and she began to exhibit some clinical symptoms on January 19, 2017, about 9 months after she was bitten on the right arm by a stray dog in a pasture, and failed to seek post-exposure prophylaxis (PEP). On January 23, the patient died, and her brain and neck skin specimens were collected, and all the specimens, including the liquid specimens collected 2 days previously, were handed to Tibet CDC and then transported to our lab at China CDC in Beijing. According to the epidemiology survey performed by the local staff with the case’s relatives, a stray dog has bitten the woman and had also bitten another person, who had died before this reported case and was given a sky burial. The sky burial master was found by the local CDC, and was given PEP.

Canine Sample: In addition to these cases, in 2012, 6 villagers in Jiali County in Naqu Prefecture (the same location as Case 1) were bitten by a stray dog. The dog was killed, vaccination was arranged for the affected villagers, and brain specimens were collected from the dog by Tibet CDC and sent to our lab for testing and confirmed to be positive [9]. This remains the only recent reported animal case in Tibet until now.

### The emergence of a new rabies lineage in China

**Fig 2:**
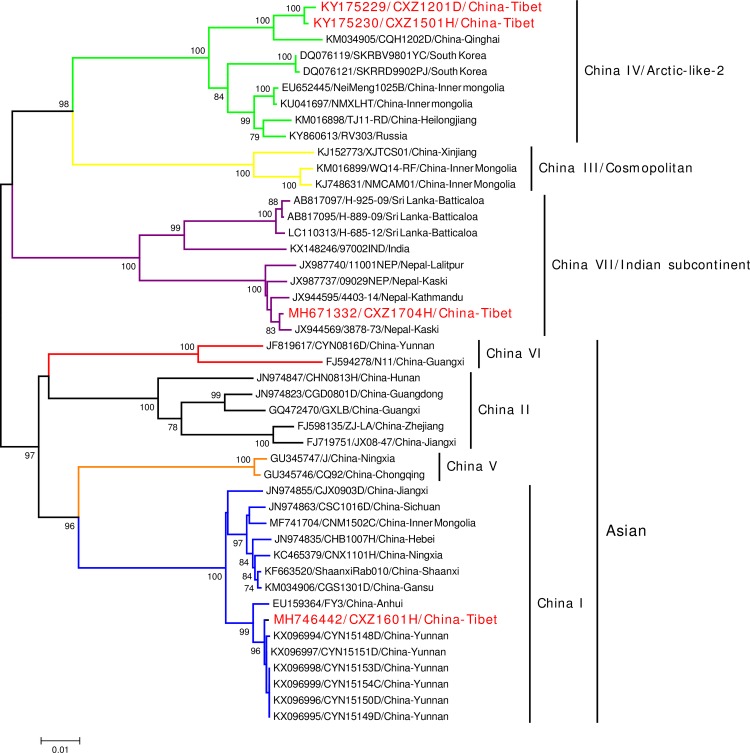
NJ tree for the three human cases and dog case from Tibet and 41 national and global reference strains, based on complete N genes. Human sample CXZ1501H collected in 2015 and canine sample CXZ1201D collected in 2012 are placed in the China IV lineage within the Arctic-like-2 lineage, but human sample CXZ1704H, collected in 2017, is placed in the Indian Subcontinent lineage with reference strains from India, Sri Lanka and Nepal, with the Chinese strain placing closest to the Nepal strains, consistent with geographical proximity. Human sample CXZ1601H collected in 2016 is placed in the China I lineage, closed with strains from Yunnan, China. Horizontal bar indicates genetic distance. Branches are colored to indicate the seven different lineages circulating in China(Blue, China I; Black, China II; Yellow, China III / Cosmopolitan; Green, China IV / Arctic-like-2; Orange, China V; Red, China VI; Purple, China VII / Indian subcontinent). Taxa are in the format (ACCESSION NO/ STRAIN/ COUNTRY-PROVINCE). The strains from Tibet are highlighted with red taxa.

The estimated phylogenetic tree is shown in Fig 2. Isolates CXZ1201D and CXZ1501H, appear to be very similar and are placed within the China IV lineage, together with another recent isolate collected from Qinghai in 2012 [9], consistent with our original hypothesis that they were a consequence of wildlife spillover, rather than associated with the current epizootic[9]. However, surprisingly, the most recently collected isolate CXZ1704H (Case 3) is evolutionarily distinct from the 6 currently known Chinese lineages [1]. The structure of the tree is consistent with the corresponding genome tree (S1 Fig).

Both figures place CXZ1704H in the Indian subcontinent clade (commonly associated with Indian and Sri Lankan isolates), rather than being placed in the Asian or Arctic-like clades with other Chinese strains[13]. The closest strain in the reference set was the isolate from Nepal [25]. Shigatse city in Tibet, which is where the patient lived, neighbors with Nepal (Fig 1).

This is the first report of an Indian subcontinent strain in Tibet and in China as a whole. We therefore placed it in a new Chinese rabies lineage, China VII.

### The dominant rabies epizootic lineage has reached Tibet

Additionally, the staff of Sichuan CDC was able to complete N gene sequencing for Case 2, using the remainder of the saliva sample. The sample is placed in the China I lineage (Fig 2), and closest to strains from Yunnan, located in southeast China (Fig 1) whose northwestern borders are adjacent to southeast Tibet. China I is the dominant lineage in the current rabies epizootic, expanding from South to North China, and has encroached into most western provinces in recent years [1,9]. Thus, China I has finally spread to Tibet, which is the last province in mainland China to report human cases.

## Discussion

Effective strategies have been developed for rabies eradication in the western hemisphere including large scale dog vaccination, controlling populations of roaming dogs and bait drops [26,27]. However, for large countries such as China, with broad geographical diversity, these strategies can aid control of an epizootic, but the magnitude of the task raises new challenges; for example, the size of the country makes widespread bait dropping unfeasible. Thus, eradicating an epizootic requires a longer term strategy. Monitoring programs play a key role in evaluating the efficacy of control strategies and identifying potential new cases in low incidence regions to halt further dissemination of the virus [3,7].

Tibet has remained virtually rabies free for almost 20 years, but recent sporadic cases have raised concerns that the current epizootic may gain a foothold in this region. The first rabies cases in 16 years was reported for a dog with rabies in Naqu Prefecture in 2012 [9] and in 2015 the first human case was reported, also in Naqu Prefecture (Fig 1).

Our previous investigations of sequences from isolates collected from the national surveillance program have established a detailed view of the dissemination of the current epizootic [1,12,17]. The current epizootic was initially associated with the China I and China II lineages, however the latter was associated with the previous epizootic and was subsequently displaced by China I as the dominant lineage [1]. Nevertheless, China II remains associated with a number of cases in high incident regions [1]. China III to VI are present at lower levels and our analyses indicate that they are associated with spillover from wildlife [9]. For example, our analysis of recent isolates from West China indicated that they were a consequence of both the current epizootic (Gansu and Ningxia) and spillover from wildlife (Tibet and Qinghai) [1,9].

Interpreted in this context, our current results indicate that CXZ1201D and CXZ1501H, placed within the China IV lineage, are associated with wildlife spillover. This is consistent both with the placing of the other recent isolates from the neighboring provinces of Ningxia and Gansu and with the current rabies situation in these provinces and Qinghai and Tibet; human cases in Gansu rose to 11 cases in 2015, Ningxia increased to 14 cases in 2014, whereas Qinghai and Tibet have only reported one or no cases each year in recent years [8].

It was therefore surprising to find that isolate from the 2017 Tibet case (CXZ1704H) is placed in a clade that is distinct from other Chinese isolates and which corresponds to the Indian subcontinent clade [13] (Fig 2). This is particularly striking as our 2013 analysis of rabies strains in China and neighbouring countries bordering South and Southwest China (i.e., Thailand, Viet Nam, Cambodia, Laos, Nepal, India, Myanmar and Bhutan) indicated that national borders appeared to be effectively halting the spread of rabies [1,12]. However, in recent years there have been many new economic trade agreements [28] to promote trade with neighboring countries. For example, Zhangmu (樟木) was the major customs port between Nepal and China, but at the end of 2014 a second customs port, Jilong (吉隆), was opened (Fig 1). However, after the earthquake in Nepal 2015, Jilong became the major customs port placing greater burden on border controls [16]. Thus, it seems this new incursion is likely a consequence of the increased trade between Nepal and China; this not only increases the likelihood of inbound rabies cases but other diseases as well. Thus, the surveillance and research of infectious diseases needs to be strengthened, including host animal control and management.

The other conclusion from this study is that effective communication between national and local CDCs is crucial for rapid response to rabies events. Case 2 was transferred to a hospital in Sichuan where they were diagnosed with rabies, the diagnosis was confirmed by laboratory testing by the Sichuan CDC, and this was reported as a confirmed rabies case to the NDRS via the Internet. Our staff in the national laboratory of China CDC can access data in NDRS, but unfortunately there was sufficient delay so that when we contacted Sichuan CDC, the patient had already died and there was insufficient sample volume for genome amplification. If there had been a protocol in place to ensure rapid communication between local and national CDCs upon identification of a positive case, more work could have been done, i.e. collecting more saliva or serum samples before death, or obtaining a brain sample after death. This is a focus of a current review of the infrastructure between national and local CDCs.

Fortunately, the N gene of Case 2 was obtained from the limited saliva sample. Surprisingly, it belongs to China I lineage (Fig 2), which indicates the dominate lineage in the current rabies epizootic has expanded into Tibet, from neighboring Yunnan, which in recent years has experienced a reemergence of dog rabies [29]. However, given that Tibet is a vast but sparsely populated territory, it is unlikely this first case will be followed by a rapid increase in cases, giving us the opportunity to ensure further controls methods are in place to halt further spread of cases.

Our ongoing surveillance of the China rabies epizootic highlights the benefits of combining surveillance and phylogenetic data, as it presents a more complete picture and helps to better interpret the epidemic situation [1,9,17,18,30]. Nevertheless, our findings to date indicate the geographical distribution of rabies, as well as the virus lineages in China are changing. We believe more comprehensive, timely and specific surveillance will be needed to adapt to these changes to continue to reduce the number of rabies cases in the country.

## Supporting information

S1 Table Chinese and worldwide rabies N gene or genome sequences used in this study. (XLSX)

S1 Fig NJ tree with rabies genomes for Case 1, Case 3 and the dog case from Tibet in Fig 1 and 41 global reference strains that are representative of the main global lineages. Horizontal bar indicates genetic distance. Branches are colored to indicate the seven different lineages circulating in China (Blue, China I; Black, China II; Yellow, China III / Cosmopolitan; Green, China IV; Orange, China V; Red, China VI; Purple, China VII). Taxa are in the format (ACCESSION NO/ STRAIN/ COUNTRY-PROVINCE). The strains from Tibet are highlighted with red taxa.
(TIF)
