# Supplementary figures and images for: The reemergence of human rabies and emergence of an Indian subcontinent lineage in Tibet, China
Source: PLoS Negl Trop Dis. 2019 Jan 14;13(1):e0007036. doi: 10.1371/journal.pntd.0007036 (PMC6349412; doi:10.1371/journal.pntd.0007036)

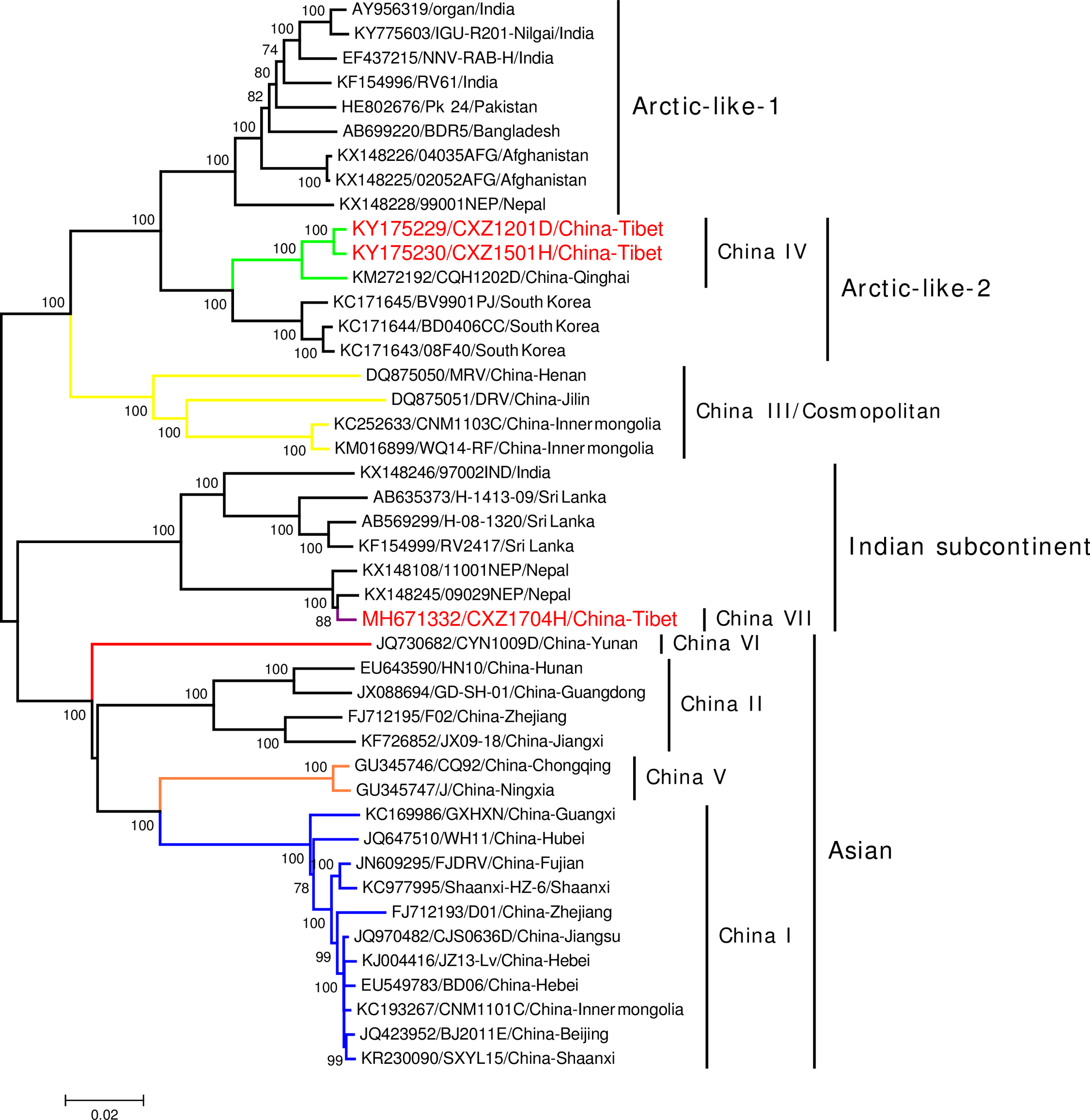

Supplement: S1 Fig — Horizontal bar indicates genetic distance. Branches are colored to indicate the seven different lineages circulating in China (Blue, China I; Black, China II; Yellow, China III / Cosmopolitan; Green, China IV; Orange, China V; Red, China VI; Purple, China VII). Taxa are in the format (ACCESSION NO/ STRAIN/ COUNTRY-PROVINCE). The strains from Tibet are highlighted with red taxa. (TIF) [file pntd.0007036.s002.tif]
